# Persistent Tenosynovitis, Steroid Dependency and a Hyperpigmented Scaly Macular Rash in a Child With Juvenile Idiopathic Arthritis

**DOI:** 10.7759/cureus.11208

**Published:** 2020-10-27

**Authors:** Eleni Maria Papatesta, Lydia Kossiva, Maria Tsolia, Despoina Maritsi

**Affiliations:** 1 Pediatrics, Panagiotis & Aglaia Kyriakou Children's Hospital, Athens, GRC

**Keywords:** blau syndrome, canakinumab, nod2, il-1, adalimumab

## Abstract

Blau syndrome is a rare autoinflammatory disease, characterized by granulomatous symmetric arthritis, skin rash and uveitis. It is caused by mutations in the CARD15/NOD2 gene, which is a significant part of innate immunity. We describe the case of a patient with Blau syndrome, initially misdiagnosed as juvenile idiopathic arthritis. Genetic analysis showed R334Q mutation in the NOD2 gene that is known to be linked to Blau syndrome. Our patient was successfully treated with the IL-1β blocking agent canakinumab, with clinical and laboratory remission without any adverse effects. To our knowledge this is one of the rare cases of Blau syndrome successfully treated with canakinumab. After moving abroad, canakinumab was discontinued and she was treated with adalimumab instead. Change in her treatment resulted in a relapse of her disease. Prompt recognition of Blau syndrome and the optimal treatment, are vital for the prevention of severe sequelae such as vision loss and joint deformities. Canakinumab constitutes a promising therapeutic approach for Blau syndrome and requires further investigation.

## Introduction

Blau syndrome is a rare dominant autosomally-inherited autoinflammatory disease, first described in 1985 by Edward Blau. The classic triad characterizing this syndrome is granulomatous symmetric arthritis, skin rash, and uveitis. It usually affects children under 4 years old [1,2]. However, not all manifestations appear simultaneously. It is related with mutations in the CARD15/NOD2 gene, mapped on chromosome 16q12, which plays a pivotal role in pathogen recognition and innate immune response [3,4]. Although numerous mutations have been reported to date, the two most frequent mutations linked to Blau syndrome are R334W and R334Q [5,6].

## Case presentation

We describe the case of a patient with Blau syndrome, who was initially diagnosed as juvenile idiopathic arthritis, and was afterwards successfully treated with the IL-1β blocking agent canakinumab. To our knowledge, this is one of the rare cases of Blau syndrome successfully treated with canakinumab [7-9].

A 7-year-old Caucasian female presented to our clinic with the diagnosis of juvenile idiopathic arthritis. Disease onset was reported at 2 years of age. There was no hereditary family disease of note. At the age of 2 years old, she presented with high fever and swelling of knees, ankles, wrists and metacarpophalangeal joints bilaterally, associated with tenosynovitis. Laboratory tests revealed severe anemia (Hb 5.8gr/dl), elevated WBC 30000/μL (N 54%, L 35%, M 8%), elevated CRP 128mg/l and an elevated erythrocyte-sedimentation-rate (ESR) of 59mm/h. The patient was hence diagnosed with polyarticular juvenile idiopathic arthritis and her treatment included methotrexate, etanercept and prednisolone with only mild alleviation of symptoms, upon steroid withdrawal. 

When she was referred to our clinic, on physical examination, besides being cushingoid, she manifested severe swelling and pain of wrists, elbows, ankles and knees; the range of movement was mildly affected. There was also noticeable tenosynovitis throughout. Laboratory exams were as follows: WBC 12600/μL (N 67%, L 22%, M 9%), CRP 10mg/l and ESR 40mm/h. The opthalmology exam was normal. Following discontinuation of etanercept, the patient was started on tocilizumab for 4 months, which led to insignificant clinical improvement. At that point, she presented with intermittent episodes of quotidian fever and deterioration of her laboratory and physical findings. They were considered a relapse of her condition, and she was therefore admitted. During hospitalization, she developed an erythematous maculopapular scaly rash of cervical and axillary regions, which over the days became pigmented. Her mother then recalled that during early childhood she presented recurrent episodes of a similar rash. Meanwhile, slit-lamp examination revealed uveitis. Considering the polyarticular non-deforming involvement, excessive tenosynovitis and the presence of the scaly maculopapular rash, the diagnosis of juvenile idiopathic arthritis was clearly questioned. Next generation sequencing (NGS) gene panel analysis linked to autoinflammatory diseases was performed, showing an heterozygous c.1001G>A, p.Arg334Gln mutation (R334Q) in NOD2. As previously mentioned, this mutation is associated with Blau syndrome. In combination with three methylprednisolone pulses and gradual steroid reduction, we initiated therapy with canakinumab leading to a prompt clinical improvement of her fever, arthritis, tenosynovitis, rash and uveitis accompanied by a normalization of the laboratory markers. Since our patient did not achieve remission with etanercept and taking into consideration that Blau syndrome is an autoinflammatory disease, canakinumab was regarded as the best therapeutic option.

After 12 months on canakinumab monotherapy, she was in complete clinical and laboratory remission, while her quality of life was substantially improved. During the entire care period, neither serious adverse event, nor increased incidence of acute infections was reported.

After she had to move with her family to another European country, her therapy with canakinumab was discontinued (due to unavailability), and she was treated with adalimumab. She subsequently presented recurrence of her disease, with fever, reappearance of arthritis and worsening of her laboratory findings. At the moment she is steroid dependent, and her treatment includes adalimumab and methotrexate.

## Discussion

There are no official guidelines regarding the therapy of Blau syndrome, due to its infrequency and the diversity of its clinical presentation and only case series have been published [9,10]. Apart from corticosteroids and methotrexate, the biological agents most frequently used are TNFi (tumor necrosis factor inhibitors), with inconsistent therapeutic response [11,12]. They appear to be more effective in controlling articular disease, whereas ocular disease seems more resistant [10,13]. Etanercept and adalimumab have insufficient disease control in numerous patients, with the underlying reasons for this remain unclear [5,11,13,14].

To our knowledge, there are a few cases of patients who were successfully treated with canakinumab in literature. Canakinumab, a human anti-ILβ monoclonal antibody, is approved for the treatment of other autoinflammatory diseases, such as cryopyrin-associated periodic syndromes (CAPS), tumor necrosis factor receptor associated periodic syndrome (TRAPS), hyperimmunoglobulin D syndrome (HIDS) and familial Mediterranean fever (FMF) [15]. Blau syndrome is an autoinflammatory disease caused by a mutation in the NOD2 gene, which belongs to the NOD like receptor family (NLR). NOD2 is an intracellular receptor of bacterial petidoglycanes, such as muramyl dipeptide (MDP). After their binding, the signaling pathway of NF-kb is activated, resulting to the transcription of proinflammatory cytokines. The believed cytokines involved are mainly the IL-1β, like in other autoinflammatory diseases [16]. In a study from Arostegui et al, plasma cytokines of patients with Blau syndrome were analyzed and an increase in IL-6, TNF and a smaller increase in IL-1β was observed in patients compared to controls, which normalized after treatment with anakinra [6]. Furthermore, mutations in another NLR family member, NLR family pyrin domain containing 3 (NLRP3), are responsible for cryopyrinopathies, via the activation of the caspase 1 inflammasome and the subsequent excess production of IL-1β. NOD2 receptors seem to also participate in the inflammasome complex [17]. In addition, Simonini et al found upregulation of transcripts associated with the innate immune system in patients with Blau syndrome and normalization of the majority of these variations after canakinumab was initiated [7]. Based on the above reports, therapeutic options which include IL-1 blockage have been reported in the literature with promising results [6,7,9]. Nevertheless, there are studies that haven’t exhibited the same outcomes. Increased levels of IL-1β were not observed after analysis of cytokine plasma levels of patients with Blau syndrome, and treatment with anakinra was unsuccessful [14,16]. 

## Conclusions

In patients with treatment resistant polyarticular arthritis and severe tenosynovitis, especially in the presence of atypical skin rash or late ocular involvement, Blau syndrome should be suspected and gene analysis should be performed. Early recognition of the disease leads to prevention of serious complications. Moreover, our patient’s clinical improvement following treatment with IL-1 blocking agent, has been similarly reported in the literature indicates that canakinumab (an IL-β receptor antagonist) represents a promising therapeutic option for Blau syndrome and hence requires further investigation.
